# To live is well but to live well is better: venetoclax combination therapy and quality-of-life in acute myeloid leukemia

**DOI:** 10.1038/s41408-022-00672-y

**Published:** 2022-04-25

**Authors:** Naseema Gangat, Ayalew Tefferi

**Affiliations:** Division of Hematology, Mayo Clinic, Rochester, MN United States

**Keywords:** Leukaemia, Chemotherapy

## Venetoclax combination therapy and acute myeloid leukemia

The FDA approval of venetoclax in combination with hypomethylating agents (azacitidine or decitabine) or low-dose cytarabine has offered renewed hope for elderly/unfit patients with newly diagnosed acute myeloid leukemia (AML). In the pivotal Phase III VIALE-A and VIALE-C studies, complete response rates were superior with venetoclax combination therapy compared to azacitidine (66% vs 28%), and low-dose cytarabine alone (48% vs 13%); moreover, overall survival was prolonged at 14.7 and 8.4 with venetoclax plus azacitidine and low-dose cytarabine, respectively [1, 2]. Major toxicities included grade 3 or higher thrombocytopenia (45%/45%), neutropenia (42%/47%), and febrile neutropenia (42%/32%) in the respective VIALE-A and C studies; [1, 2] additionally, 44% of patients receiving azacitidine plus venetoclax experienced nausea [1]. Since health-related quality of life, particularly physical functioning is generally poor in geriatric patients with AML ineligible for intensive chemotherapy and goals of therapy are palliative [3], a global assessment of the patient’s perception of the physical and psychosocial impacts of leukemia-directed therapies is imperative for informed therapeutic decisions.

## Health-related quality-of-life with venetoclax combination therapy

According to the recently published health-related quality-of-life analysis by Pratz and colleagues, venetoclax combination therapies have the potential to positively impact symptoms and physical functioning in elderly and/or unfit patients with AML [4]. The particular study presents patient-reported outcomes of AML patients enrolled on the VIALE-A and C trials, through standard cancer assessment tools namely Patient-Reported Outcomes Measurement Information System (PROMIS) Cancer Fatigue Short Form 7a (Fatigue), the European Organization for Research and Treatment of Cancer quality of life questionnaire, EORTC QLQ-C30 global health status (GHS)/QoL and physical functioning [PF] subscales, and the EQ-5D-5L health status visual analog scale (VAS) [4]. In the current work which focused on time to functional deterioration, a significantly longer time to deterioration was observed in patients receiving venetoclax plus azacitidine as opposed to azacitidine monotherapy (9.7 vs 6.2 months and 10.7 vs 3.9 months, per EORTC QLQ-C30 PF and EQ-5D-5L VAS, respectively) [4]. Similarly, venetoclax in combination with low-dose cytarabine yielded substantial improvements in functionality in all assessed measures when compared to cytarabine alone. Furthermore, a higher proportion of patients treated with venetoclax plus azacitidine (43%) as opposed to azacitidine alone (35%) or venetoclax and low-dose cytarabine (32%) vs low-dose cytarabine (18%) experienced improvements in GHS/QoL with the majority of patients (≥65%) reporting improvements by cycle 4 [4]. As expected, patients achieving complete response or complete response with incomplete count recovery (CR/CRi) with venetoclax + azacitidine, experienced a longer time to deterioration for GHS/QoL and health status (VAS) (21.3 months vs 16.6 months with azacitidine alone). Importantly, time to deterioration remained similar among patients >75 years of age regardless of the treatment regimen, suggesting that the addition of venetoclax did not incur negative impacts on quality of life [4]. Finally, a sensitivity analysis confirmed the preservation of quality of life with venetoclax combination therapy, however the inclusion of disease progression, relapse, and death as endpoints resulted in shortened median times in all groups [4].

Although the EORTC QLQ-C30 has been validated in patients with cancer and utilized in a third of prior AML studies, it is neither disease nor treatment-specific [5, 6]. In addition, pre-defined deterioration thresholds of ≥10, 7, and 5 points in EORTC QLQ-C30, EQ-5D-5L VAS, and PROMIS Fatigue, respectively, may not always capture clinically meaningful changes in function. To that end, an AML-specific quality of life tool (AML-QOL) was recently developed which incorporates the experiences of patients receiving intensive and non-intensive chemotherapy; furthermore, the aforementioned tool has been prospectively validated in patients receiving intensive chemotherapy and found to be highly consistent, reliable and valid when compared to the EORTC QLQ-C30 [7]. Nonetheless, further investigations are required to not only determine the most clinically useful QOL tool for AML with the inclusion of variables related to number and length of hospitalizations but also to identify the optimal timing of assessments.

This study has important practical implications for patient-centric care. Foremost, the current report highlights the health-related quality-of-life benefits reaped with venetoclax-based therapy in elderly/unfit AML patients, regardless of remission status. Given the palliative intent of therapy, preservation of functioning is paramount for the treatment regimen to be acceptable to elderly patients, hence the findings regarding the absence of a negative impact of venetoclax-based therapy on quality of life in patients over 75 years of age, are reassuring. Second, as one is faced with similarly efficacious therapeutic choices with the availability of FLT3 and IDH1/2 inhibitors, a comparative assessment of quality-of-life measures may enable informed therapeutic decisions. However, it should be brought to attention that drug labels for several of the recently approved AML therapies including decitabine, FLT3 inhibitors (midostaurin/gilteritinib), IDH1/2 inhibitors (ivosidenib/enasidenib) and venetoclax, lack information on patient-reported outcomes. The question remains on whether results from the current study are generalizable to patients treated in routine practice since both VIALE-A and C trials were limited to patients over 75 years of age with ECOG performance status ≤2. Therefore, these findings require prospective validation in real-world series preferably utilizing AML-specific patient-reported outcome measures. Additional limitations of the study include unreported changes in quality of life since assessments were performed every other cycle (month) and the attrition rate was high beyond earlier cycles of therapy, which deserves attention given the continuous nature of venetoclax-based therapy.

## Future considerations

**Fig. 1:**
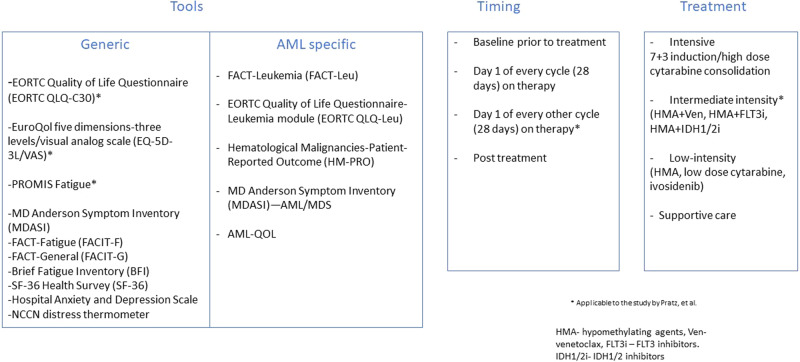
Considerations for patient-reported outcome measures in acute myeloid leukemia; 3Ts (tools/timing/treatment).

In summary, venetoclax combination therapy has refreshingly changed the treatment paradigm for elderly/unfit AML by not only adding years to life but also life to years. However, the incorporation of patient-reported outcomes in AML is met with unique challenges, especially with respect to the heterogenous assessment tools utilized, and calls for immediate identification of disease and treatment-specific consensus instrument (Fig. 1) [8]. Furthermore, longitudinal health-related quality of life evaluations should be routinely conducted in clinical practice at optimal time points both during and after cessation of treatment in patients with AML receiving venetoclax combination therapy.

## References

[1] DiNardo CD, Jonas BA, Pullarkat V, Thirman MJ, Garcia JS, Wei AH (2020). Azacitidine and venetoclax in previously untreated acute myeloid leukemia. N Engl J Med.

[2] Wei AH, Montesinos P, Ivanov V, DiNardo CD, Novak J, Laribi K (2020). Venetoclax plus LDAC for newly diagnosed AML ineligible for intensive chemotherapy: a phase 3 randomized placebo-controlled trial. Blood.

[3] Forsythe A, Kwon CS, Bell T, Smith TA, Arondekar B (2019). Health-related quality of life in acute myeloid leukemia patients not eligible for intensive chemotherapy: results of a systematic literature review. Clinicoecon Outcomes Res.

[4] Pratz KW, Panayiotidis P, Recher C, Wei X, Jonas BA, Montesinos P, et al. Venetoclax combinations delay the time to deterioration of HRQol in unfit patients with acute myeloid leukemia. Blood Cancer J. 2022; in press. 10.1038/s41408-022-00668-8 PMC9021259 35443742

[5] Loh KP, Abdallah M, Kumar AJ, Neuendorff NR, Dahiya S, Klepin HD (2019). Health-related quality of life and treatment of older adults with acute myeloid leukemia: a young international society of geriatric oncology review paper. Curr Hematol Malig Rep..

[6] Stauder R, Lambert J, Desruol-Allardin S, Savre I, Gaugler L, Stojkov I (2020). Patient-reported outcome measures in studies of myelodysplastic syndromes and acute myeloid leukemia: Literature review and landscape analysis. Eur J Haematol.

[7] Buckley SA, Halpern AB, Othus M, Jimenez-Sahagun D, Walter RB, Lee SJ (2020). Development and validation of the AML-QOL: a quality of life instrument for patients with acute myeloid leukemia. Leuk Lymphoma.

[8] Buckley SA, Kirtane K, Walter RB, Lee SJ, Lyman GH (2018). Patient-reported outcomes in acute myeloid leukemia: where are we now?. Blood Rev.

